# Rapamycin modulates pulmonary pathology in a murine model of *Mycobacterium tuberculosis* infection

**DOI:** 10.1242/dmm.049018

**Published:** 2021-10-26

**Authors:** Kamlesh Bhatt, Madhuri Bhagavathula, Sheetal Verma, Graham S. Timmins, Vojo P. Deretic, Jerrold J. Ellner, Padmini Salgame

**Affiliations:** 1 Center for Emerging Pathogens, Department of Medicine, Rutgers New Jersey Medical School, Newark, NJ 07103, USA; 2 Department of Pharmaceutical Sciences, University of New Mexico, Albuquerque, NM 87131, USA; 3 Autophagy Inflammation and Metabolism (AIM) Center of Biomedical Research Excellence University of New Mexico Health Sciences Center, Albuquerque, NM 87131, USA; 4 Department of Molecular Genetics and Microbiology, University of New Mexico Health Sciences Center, Albuquerque, NM 87131, USA

**Keywords:** Tuberculosis, Host-directed therapy, Rapamycin, Inflammation

## Abstract

Tuberculosis (TB) treatment regimens are lengthy, causing non-adherence to treatment. Inadequate treatment can lead to relapse and the development of drug resistance TB. Furthermore, patients often exhibit residual lung damage even after cure, increasing the risk for relapse and development of other chronic respiratory illnesses. Host-directed therapeutics are emerging as an attractive means to augment the success of TB treatment. In this study, we used C3HeB/FeJ mice as an experimental model to investigate the potential role of rapamycin, a mammalian target of rapamycin inhibitor, as an adjunctive therapy candidate during the treatment of *Mycobacterium tuberculosis* infection with moxifloxacin. We report that administration of rapamycin with or without moxifloxacin reduced infection-induced lung inflammation, and the number and size of caseating necrotic granulomas. Results from this study strengthen the potential use of rapamycin and its analogs as adjunct TB therapy, and importantly underscore the utility of the C3HeB/FeJ mouse model as a preclinical tool for evaluating host-directed therapy candidates for the treatment of TB.

## Abstract (precis)

**Summary:** Rapamycin, an mTOR inhibitor, with or without moxifloxacin, reduces lung inflammation and the number and size of caseating necrotic granulomas in *Mycobacterium tuberculosis*-infected C3HeB/FeJ mice.

## INTRODUCTION

Tuberculosis (TB), caused by *Mycobacterium tuberculosis* (Mtb), remains a significant global burden. Worldwide, an estimated 10.0 million people developed TB disease in 2019, and there were an estimated 1.4 million TB deaths (Chakaya et al., 2021). Several drug regimens are available for the treatment of TB; however, the duration of the regimen is lengthy (6 months for drug-susceptible TB and more than 18 months for drug-resistant TB). Moreover, these treatment modalities are often associated with severe side effects (Hosford et al., 2015; Sayada, 2010). Patients that complete treatment regimens successfully still frequently experience permanent lung damage due to aberrant inflammation in response to Mtb (Pasipanodya et al., 2010; Wallis and Hafner, 2015; Zumla et al., 2015). For example, in patients who have completed treatment for multidrug resistant disease (mean, 21 months), the first forced expiratory volume (FEV_1_) was only 63% of the predicted value, indicating significant loss of lung function (de Vallière and Barker, 2004).

To circumvent the complications associated with microbial drug resistance and to mitigate Mtb-induced inflammatory damage to the host, investigational research has turned towards host-directed therapies (HDTs), some of which are already being tested for clinical use (Kaufmann et al., 2014; Hawn et al., 2013). Four focus areas that drive the development of novel HDTs against TB include the following: (1) improving the efficacy of anti-microbial mechanisms and decreasing the length of TB treatment; (2) reducing inflammation during TB treatment and permanent lung damage at the end of treatment; (3) improving memory immune responses against the pathogen; and (4) designing drugs that readily penetrate the tuberculous granuloma for better access to the bacilli (Young et al., 2020; Tsenova and Singhal, 2020). That said, developing a standard HDT regimen for TB is challenging as the disease presents itself as a broad spectrum, ranging from latent to incipient to subclinical disease prior to progression to clinical symptomatic TB disease (Pai et al., 2016). Yet, drugs that target core immunometabolic pathways in the host can potentially have a significant impact on the treatment outcome (Hnizdo et al., 2000; Ravimohan et al., 2018; Pasipanodya et al., 2007).

Autophagy has been widely studied for its role in protection during Mtb infection (Deretic, 2014; Gutierrez et al., 2004; Watson et al., 2012). Autophagy-competent mice have a lower lung bacterial load and reduced lung pathology compared to autophagy-deficient mice, both during Mtb Erdman and Mtb H37Rv infections (Castillo et al., 2012; Bonilla et al., 2013). These findings suggest that targeting autophagy to ameliorate chronic lung inflammation during TB treatment can potentially lead to the development of novel adjunct therapies. One of the core regulatory pathways that mediates autophagy is the mammalian target of rapamycin (mTOR). Rapamycin, a known immunosuppressive drug, binds to mammalian target of rapamycin complex 1 (mTORC1), blocking its downstream signaling and resulting in the induction of autophagy (Jung et al., 2010; Noda and Ohsumi, 1998; Dumont and Su, 1995; Thomson et al., 2009). Rapamycin treatment-induced autophagy decreases intracellular mycobacterial survival, as seen in murine bone marrow-derived macrophages and primary human monocytes (Gutierrez et al., 2004). A similar reduction in intracellular bacterial load was also reported in THP1 macrophages treated with rapamycin (Gupta et al., 2014). We (Verma et al., 2019) and others (Harper et al., 2012; Kramnik et al., 2000; Pan et al., 2005; Ordonez et al., 2016; Irwin et al., 2015; Ong et al., 2014) have shown that C3HeB/FeJ mice develop caseating necrotic granulomas in the lung during chronic Mtb infection, closely resembling the pulmonary pathology seen in TB patients. Hence, we employed this mouse strain as our experimental model to study the effect of rapamycin treatment on lung inflammation and immunopathological disease, and the potential use of rapamycin as an adjunct HDT candidate during TB treatment.

In this study, we observed that treatment with rapamycin alone or as an adjunct therapy led to a significant reduction in lung inflammation and immunopathology in C3HeB/FeJ mice during Mtb infection. This treatment did not have any substantial impact on lung and spleen bacterial burden. Overall, these findings highlight the use of C3HeB/FeJ mice as a valuable model system to evaluate HDT candidates targeted against infection-induced pathology and further emphasize the opportunity that adjunct therapeutic agents, such as rapamycin and its analogs, present in modulating disease outcomes in a real-world setting.

## RESULTS

### Rapamycin treatment alone decreases immunopathology in Mtb-infected mice

The addition of rapamycin to *in vitro* macrophage cultures induces autophagy and results in the significant control of intracellular bacterial replication (Gutierrez et al., 2004). Therefore, we investigated whether rapamycin treatment would similarly reduce bacterial burden *in vivo* in Mtb-infected mice. The phosphorylation of ribosomal protein S6 is widely used as an indicator of mTOR activity. Therefore, to confirm that rapamycin was active in the lungs of infected mice, lung tissue sections from Mtb-infected mice fed either Eudragit or microencapsulated rapamycin (eRAPA) diets for 2 weeks were stained with antibodies reactive against phosphorylated ribosomal protein S6. eRAPA treatment led to a significant reduction in phospho-S6 expression in bronchial epithelial lining compared to control animals treated with Eudragit (Fig. S1). This indicates that the mTORC1 pathway is inhibited in eRAPA-treated animals and confirms that rapamycin is active in Mtb-infected lungs.

We first evaluated whether rapamycin alone would reduce bacterial burden and, in addition, alleviate disease pathology. Mice were started on a diet of Eudragit only or Eudragit with rapamycin (eRAPA) at different time points following Mtb infection. To rule out the possibility of discrepancies arising from differential rapamycin uptake, we also assessed the serum concentration of rapamycin in uninfected animals and found very little variance in steady-state serum rapamycin levels (Table S1).

We found that when mice were administered eRAPA starting at week 2 post Mtb infection, all five mice in the eRAPA-treated group had to be euthanized at ∼2 weeks as they showed signs of becoming moribund. Gross observation of the lungs indicated severe immunopathology. This suggests that initiating rapamycin treatment early on likely affects the development of T cell immunity, and so this treatment regimen was not used later for testing the adjunct activity of rapamycin with moxifloxacin. In the succeeding experiment, eRAPA was administered for 4 weeks starting at week 4 post Mtb infection and referred to as regimen 1 (Fig. S2). Evaluation of bacterial numbers in the lung at the end of treatment (8 weeks following Mtb infection) showed that, contrary to what was observed *in vitro*, eRAPA-treated mice actually exhibited a small increase in bacterial burden in lung and spleen, albeit not significant (Fig. S3).

**Fig. 1.**
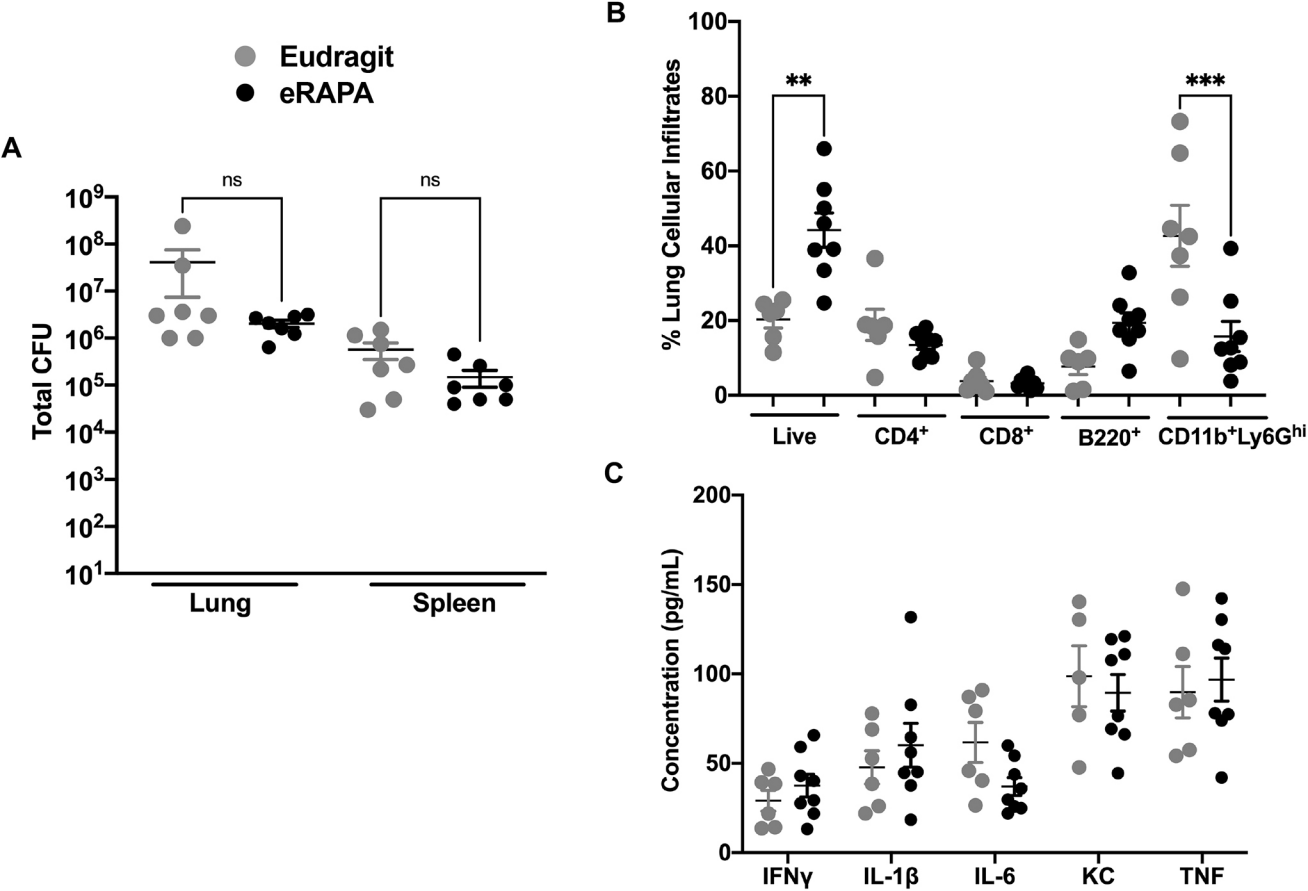
**Modulation of bacterial burden and pulmonary cell infiltration in mice treated with eRAPA alone during chronic Mtb infection.** (A) C3HeB/FeJ mice were infected with ∼50-100 CFUs of Mtb Erdman. At 7 weeks post Mtb infection, mice were divided into two groups and fed either Eudragit (microencapsulation polymer) control diet or eRAPA diet for 2 weeks. Thereafter, the animals were euthanized and whole spleen and superior lung lobe, were homogenized for estimating the bacterial burden. (B) Single-cell lung preparations were obtained, and the Trypan Blue exclusion method was used to calculate the total live cells in the lungs of the mice at 9 weeks post infection. Lung cells from both groups of mice were also surface stained with directly conjugated antibodies to quantify immune cell subsets by flow cytometry. Lung lysates were obtained from both groups of mice on either Eudragit or eRAPA diets after homogenizing lung tissue in 1 ml PBS and 2× protease inhibitor (Thermo Fisher Scientific). (C) Levels of different immune mediators were evaluated in filtered cell-free lysates using a multiplex MSD platform. The Eudragit group had *n*=7 mice, and the eRAPA group had *n*=8 mice. Data are mean±s.e.m. **P<0.005; ***P<0.0001; ns, not significant (one-way ANOVA with Kruskal–Wallis test).

**Fig. 2.**
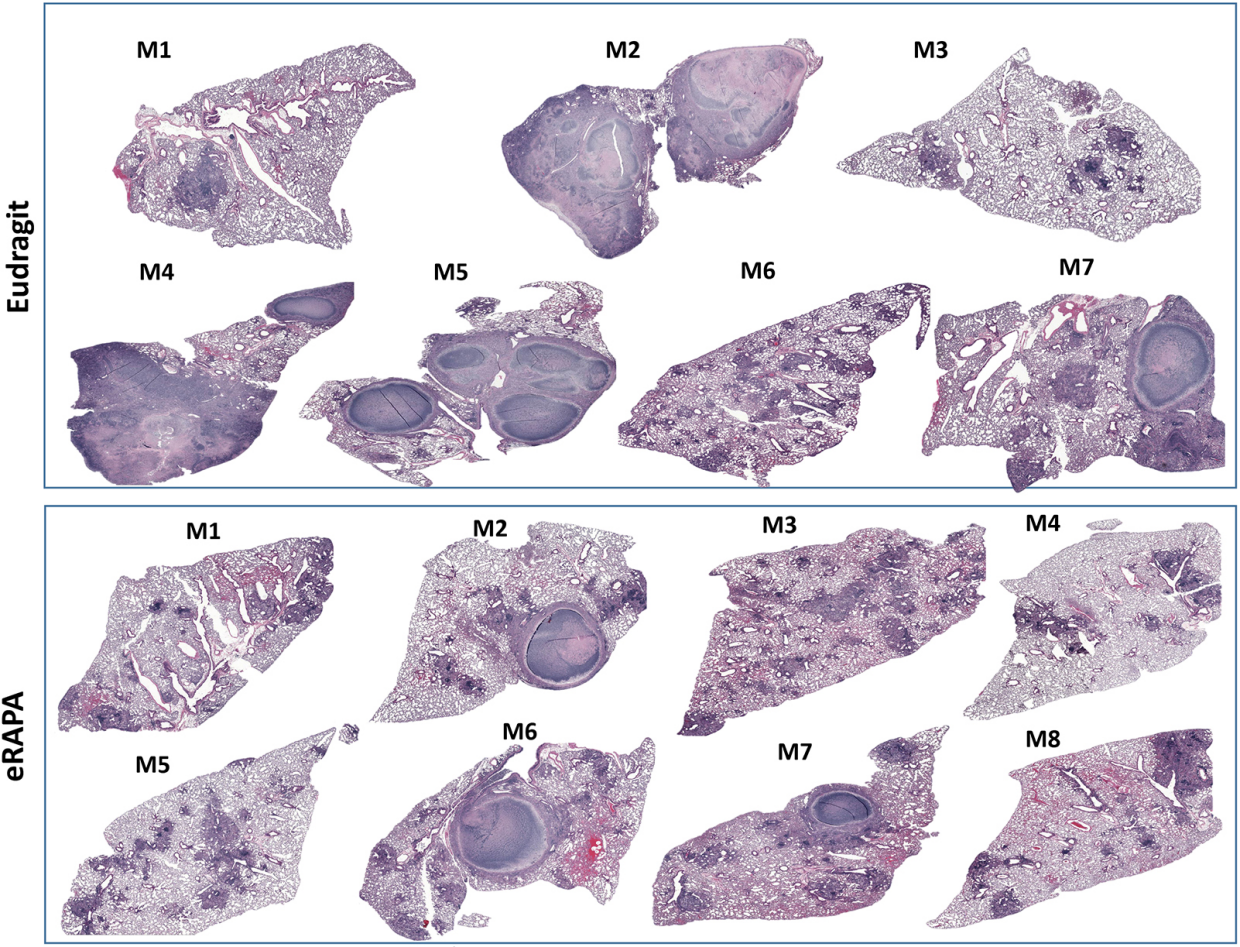
**Differential granulomatous response and necrotic lesion development in mice treated with eRAPA alone during chronic Mtb infection.** Mice were infected with low dose (∼50-100 CFUs) of Mtb Erdman, and at 7 weeks post Mtb infection, they were either fed a Eudragit diet or an eRAPA diet for 2 weeks. Corresponding mosaic H&E images of lung tissue from each animal in the two groups are shown here. These images were created using Surveyor software with Turboscan by 20× objective imaging.

Initiation of rapamycin treatment in the chronic phase most closely resembles the clinical situation in which HDT would be initiated during active disease. In the next regimen (regimen 2; Fig. S2), eRAPA treatment was therefore initiated in the chronic phase at week 7 post Mtb infection and administered for only 2 weeks. In this second treatment regimen, we found a trend towards decreased colony-forming units (CFUs) in eRAPA-treated mice, but compared to control mice the difference between the two groups was not statistically significant (Fig. 1A). Flow cytometric analysis showed that the percentage of live cells in lung single cell suspensions was significantly higher in the eRAPA-treated group, but the percentage of neutrophils (CD11^b^+Ly6G^hi^) was significantly lower in this group compared to the Eudragit group (Fig. 1B). Expression of interferon (IFN)γ, interleukin (IL)-1β, IL-6, CXCL1 (also known as KC) and tumor necrosis factor (TNF) was similar in the two groups (Fig. 1C). Histopathological examination of lung tissue (left lung lobe) revealed significant dampening of immunopathology in eRAPA-treated mice. As shown in Fig. 2, lungs from four of seven mice (Fig. 2, M2, M4, M5 and M7) in the Eudragit-treated group had focal-to-multifocal caseating necrotic granulomas that were encapsulated with central cores of acellular necrotic cell debris. In the eRAPA-treated mice, three of the eight mice (M2, M6 and M7) exhibited caseating necrotic granulomas but they were smaller and did not coalesce to occupy major areas of the lung (Fig. 2). Next, we determined whether rapamycin given by oral gavage would be more effective than that given in the diet. Following regimen 2, Mtb-infected mice at 7 weeks of infection were administered via gavage with either orange juice alone or rapamycin (0.04 mg/mouse) in orange juice (oRAPA) for 2 weeks. A comparison of lung histology revealed marked differences in the number of necrotic lesions and overall parenchymal inflammation between the oRAPA-treated and control groups (Fig. S4). Of the nine mice in the oRAPA-treated group, three (M3, M4 and M9) did not develop any necrotic lesions, three (M1, M5 and M7) had one or two lesions, and three (M2, M6 and M8) had more than three lesions. In contrast, all eight control mice developed necrotic lesions, with four (M4, M2, M7 and M8) exhibiting between one and two lesions, and the remaining four (M1, M2, M3 and M6) showing more than three lesions. Despite the recognized unevenness in necrotic lesion development in the C3HeB/FeJ mouse strain, the data presented so far provide firm evidence that mice receiving rapamycin exhibit a conspicuous reduction in lung inflammation and immunopathology.

### Prolonged 4-week rapamycin treatment with moxifloxacin significantly reduces immunopathology

**Fig. 3.**
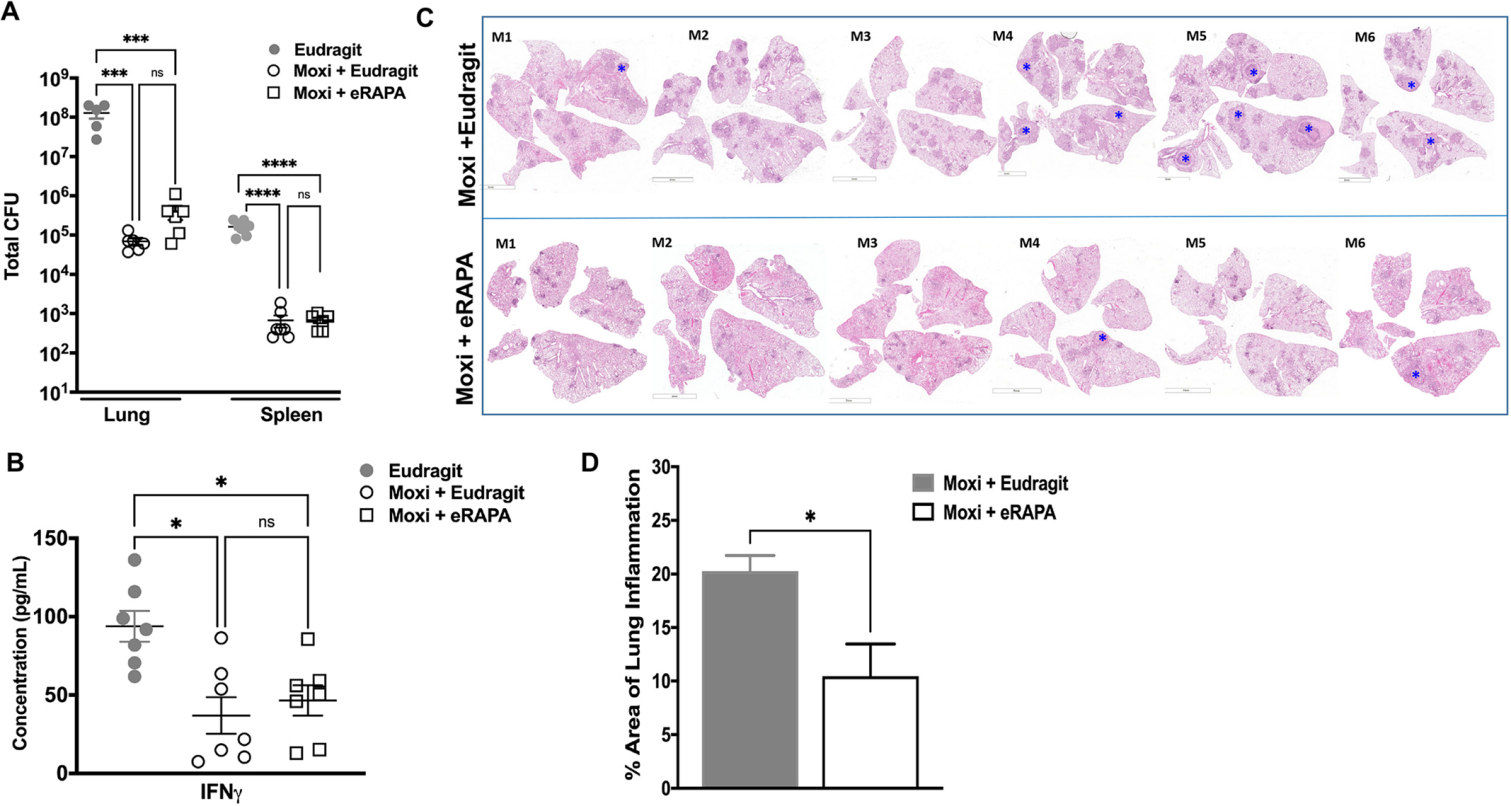
**Treatment with eRAPA and moxifloxacin during acute Mtb infection modulates lung immunopathology.** (A) Six- to 8-week-old C3HeB/FeJ mice were infected with ∼50-100 CFUs of Mtb Erdman and then categorized into three groups (Eudragit control diet with no treatment, moxifloxacin plus Eudragit and moxifloxacin plus eRAPA). These mice were treated for 4 weeks starting week 4 post infection until week 8, and then euthanized. Bacterial burden in the lungs and spleen was determined. (B) Levels of IFNγ were evaluated in filtered cell-free lysates using multiplex MSD. (C) Multilobe lung histopathological analysis was conducted, and necrotic granulomas are marked with a blue asterisk. (D) Total lung area under infection-induced inflammation was also determined. Each group included 5 or 6 mice. Data are means±s.e. Statistical significance was calculated using either an unpaired, two-tailed Student's *t*-test (two groups), one-way ANOVA (three groups) with Bonferroni's correction or one-way ANOVA with Kruskal–Wallis test (*P<0.5; ***P<0.005; ****P<0.0005; ns, not significant). Scale bars: 5 mm (C, M1, M3, M4 bottom, M5 bottom and M6); 4 mm (C, M2, M4 top and M5 top).

The data presented so far indicate that rapamycin treatment did not reduce bacterial numbers in Mtb-infected mice, but there was an appreciable decrease in immunopathology. The length of TB treatment is extremely long, i.e. 6 months for drug-susceptible TB and more than 18 months for drug-resistant TB. We argued that the ability of rapamycin to modulate immunopathology may be beneficial during TB treatment by enhancing drug efficacy. So, we next evaluated whether adjunct treatment with rapamycin would affect treatment outcome as measured by bacterial burden and immunopathology. The adjuvant activity of rapamycin with the TB drug moxifloxacin was first tested using regimen 1 (Fig. S2), which is 4 weeks of eRAPA treatment initiated at week 4 post Mtb infection in C3HeB/FeJ mice. Moxifloxacin was administered five times a week via oral gavage and given for the same length of time as eRAPA. One cohort of infected and treated mice was used for determining bacterial burden, and another for histopathological evaluation. As expected, mice receiving a diet of Eudragit and treatment with moxifloxacin exhibited a significantly reduced bacterial burden in both lungs and spleen compared to control animals that received only Eudragit (Fig. 3A). Additionally, mice receiving moxifloxacin and eRAPA showed marginally, but not significantly, higher bacterial CFUs compared to control mice receiving Eudragit and moxifloxacin (Fig. 3A). Despite the marginal increase in CFUs in the moxifloxacin plus eRAPA-treated group compared to the moxifloxacin plus Eudragit group, IFNγ expression was similar in both groups (Fig. 3B). Histopathological evaluation showed a significant decrease in disseminated lung inflammation and associated immunopathology in animals that received combination treatment of eRAPA and moxifloxacin compared to those that received moxifloxacin with the Eudragit control diet (Fig. 3C,D). Of the six mice in the group receiving moxifloxacin treatment in control Eudragit diet, four developed necrotic lesions, whereas only two of six mice in the eRAPA plus moxifloxacin group exhibited necrotic lesions. Of note, both of the mice in the eRAPA-treated group had only one lesion each.

### Two-week adjunct rapamycin treatment with moxifloxacin during the chronic phase does not substantively augment Mtb control

**Fig. 4.**
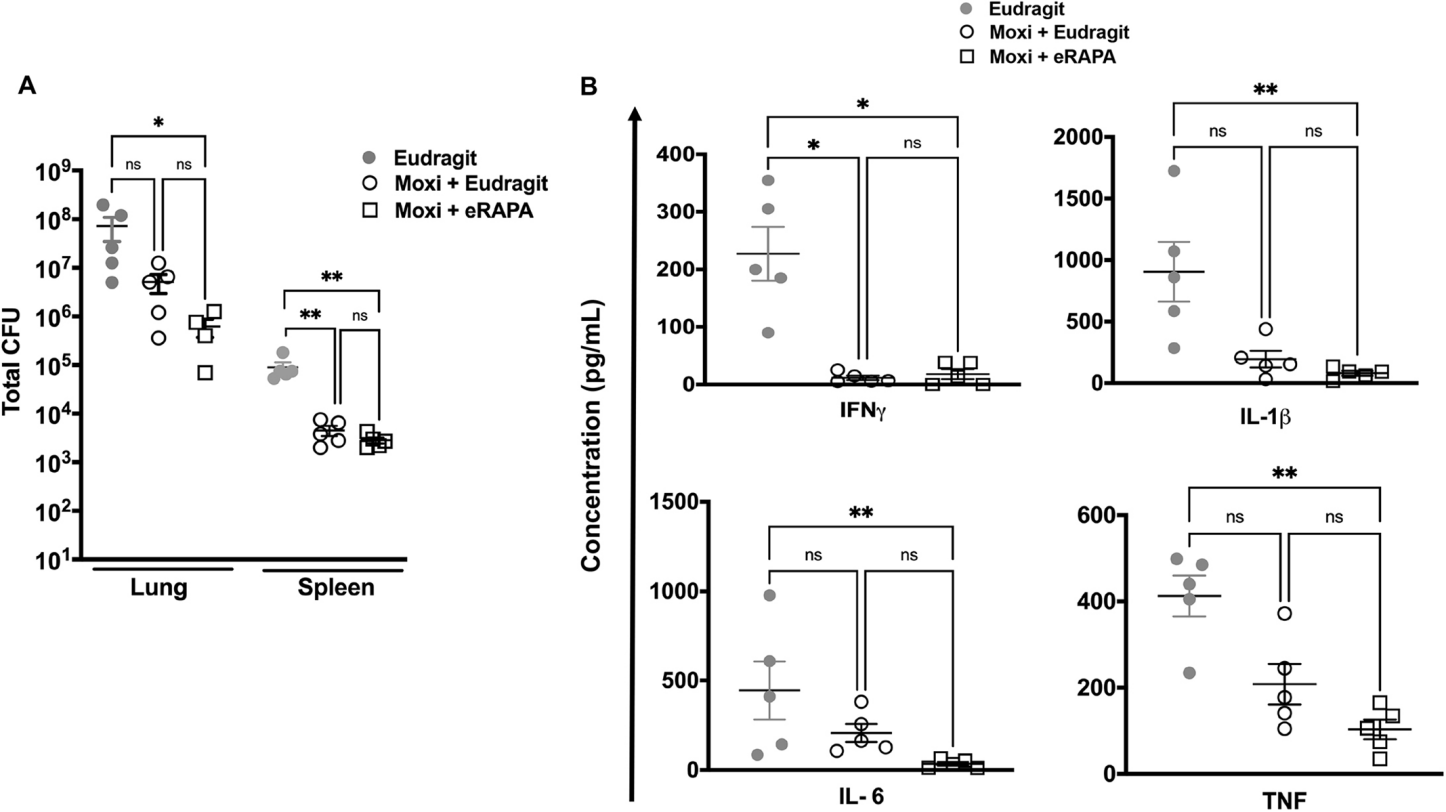
**Adjunct treatment with eRAPA and moxifloxacin during chronic infection does not significantly impact on host bacterial control and cytokine response.** (A,B) At week 7 post infection, Mtb Erdman-infected C3HeB/FeJ mice were divided into three groups (Eudragit control diet with no treatment, moxifloxacin plus Eudragit and moxifloxacin plus eRAPA) and were then treated for 2 weeks up to week 9, when they were euthanized. Bacterial burden (A) and cytokine analysis in homogenized lung lysates (B) were carried out at this time point. Five mice were included in each group. Data are means±s.e. *P<0.05; **P<0.005; ns, not significant (one-way ANOVA with Kruskal–Wallis test).

Next, we administered eRAPA to C3HeB/FeJ mice for 2 weeks, starting at a relatively chronic stage of infection (7 weeks post Mtb infection). At this time, moxifloxacin was also administered five times a week via oral gavage, while the animals were either on an eRAPA or Eudragit control diet (Fig. S2). Here too we had two cohorts, and whole lungs and spleen were harvested from euthanized animals at week 9 from one cohort to determine bacterial burden, and all lung lobes were harvested from the other cohort for histopathological evaluation. Not surprisingly, moxifloxacin administration led to a significant decrease in both lung and spleen bacterial burden in animals on either an eRAPA or Eudragit diet (Fig. 4A). Consistent with decreased CFUs, mice in both the moxifloxacin-treated groups exhibited a significant reduction in pulmonary expression of IFNγ, IL-1β, IL-6 and TNF compared to the group without moxifloxacin (Fig. 4B). Rapamycin is an immunosuppressive drug and inhibits immune responses, including CD4^+^ T cell activation (Thomson et al., 2009), and thus could significantly impact the ability of the host control Mtb infection. Although not statistically significant, lower bacterial burden in the eRAPA plus moxifloxacin group compared to the Eudragit plus moxifloxacin group (Fig. 4A), nonetheless, suggests that the immunosuppressive property of eRAPA is voided if administered later during infection.

### Two-week adjunct rapamycin treatment with moxifloxacin during chronic infection decreases infiltrative pulmonary pathology and the formation of necrotic lesions

**Fig. 5.**
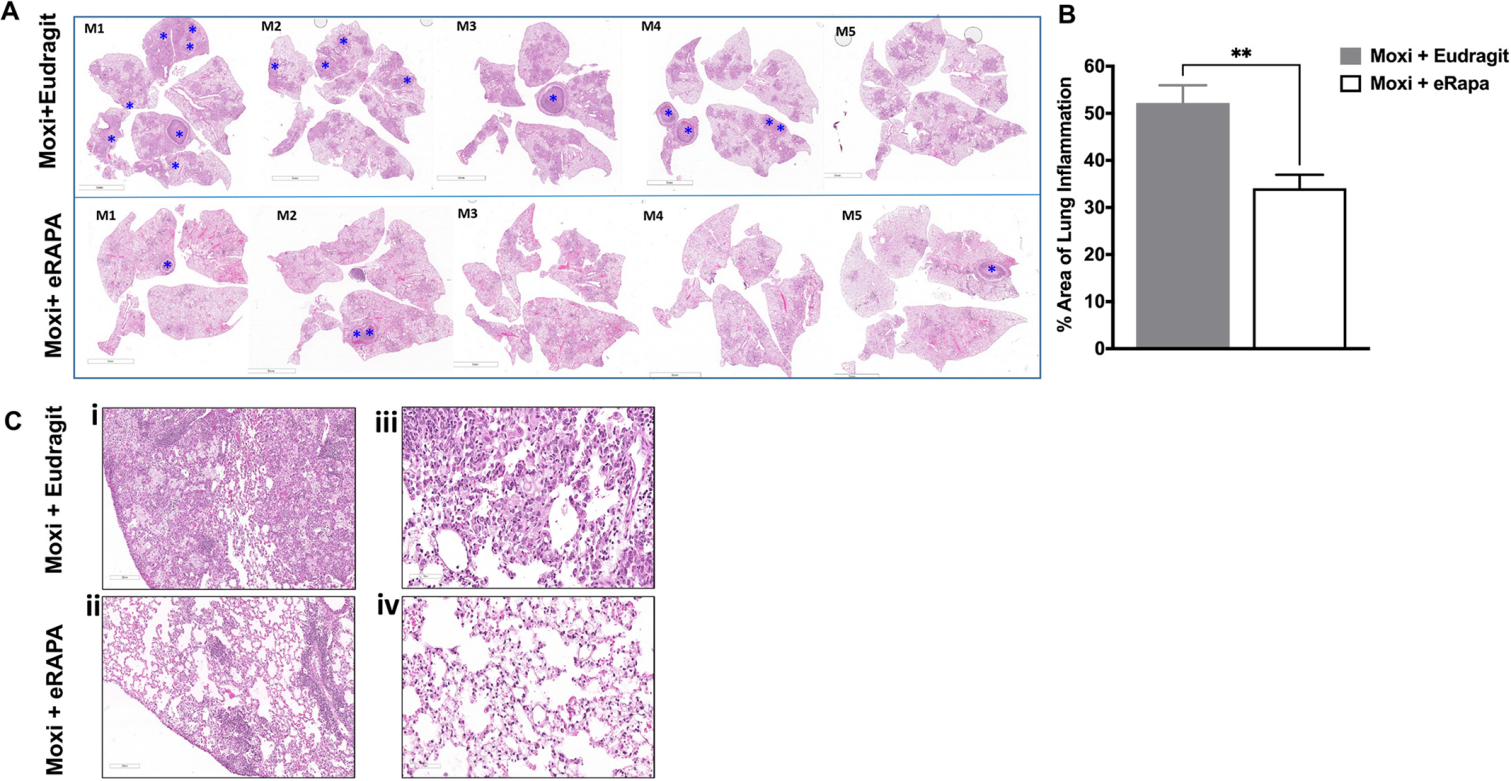
**eRAPA adjunct treatment with moxifloxacin reduces the number of necrotic lesions that develop and infiltrative pulmonary pathology.** (A) H&E images of lung tissues from Mtb-infected C3HeB/FeJ mice that received either moxifloxacin with Eudragit or moxifloxacin and eRAPA from week 7 to week 9 post infection were scanned using a Leica SCN-400 F whole-slide scanner up to 40× magnification. Images were captured at various magnifications using Aperio ImageScope to show histopathological differences between the two groups. The presence of necrotic granulomas is indicated with blue asterisks. (B) Inflamed lung area was calculated using ImageJ. (C) Differences in pulmonary pathology (Ci,Cii) and thickening of lung interstitium (Ciii,Civ) between the two groups are shown using representative images at 200 µm (10×) and 70 µm (30×) magnification. Data are means±s.e. **P<0.005 (unpaired, two-tailed Student's *t*-test). Five mice were included in each group. Scale bars: 5 mm (A); 200 µm (C).

As mice treated with eRAPA in the absence of TB drugs had reduced necrotic lesions in chronic infection, we next determined whether rapamycin given as an adjunct therapy could enhance the treatment outcome with moxifloxacin. Evaluation of Hematoxylin and Eosin (H&E)-stained lung sections from Mtb-infected animals treated with eRAPA and moxifloxacin at week 7 post infection through week 9, showed notably fewer numbers of necrotic lesions compared to those on a Eudragit and moxifloxacin diet (Fig. 5A). Of the five mice that were on a Eudragit diet and treated with moxifloxacin, three (M1, M2 and M4) had more than two lesions, one (M3) had one lesion and one (M5) had no lesions. In contrast, of the five mice receiving moxifloxacin and eRAPA, three (M1, M2 and M5) developed necrotic lesions but, of note, none of the three had more than two lesions. Quantification of lung inflammation showed a significant decrease in inflammation in the eRAPA group (Fig. 5B). Representative lung images show that the animals on moxifloxacin monotherapy and control Eudragit diet had highly infiltrative pulmonary pathology (Fig. 5Ci) compared to the animals on the eRAPA and moxifloxacin regimen (Fig. 5Cii). Animals on a Eudragit diet that received moxifloxacin exhibited significant thickening of the lung interstitium, likely due to cellular infiltration (Fig. 5Ciii). In mice that received adjunct treatment with eRAPA, there were significantly more open alveolar spaces with less inflamed alveolar septae (Fig. 5Civ).

**Fig. 6.**
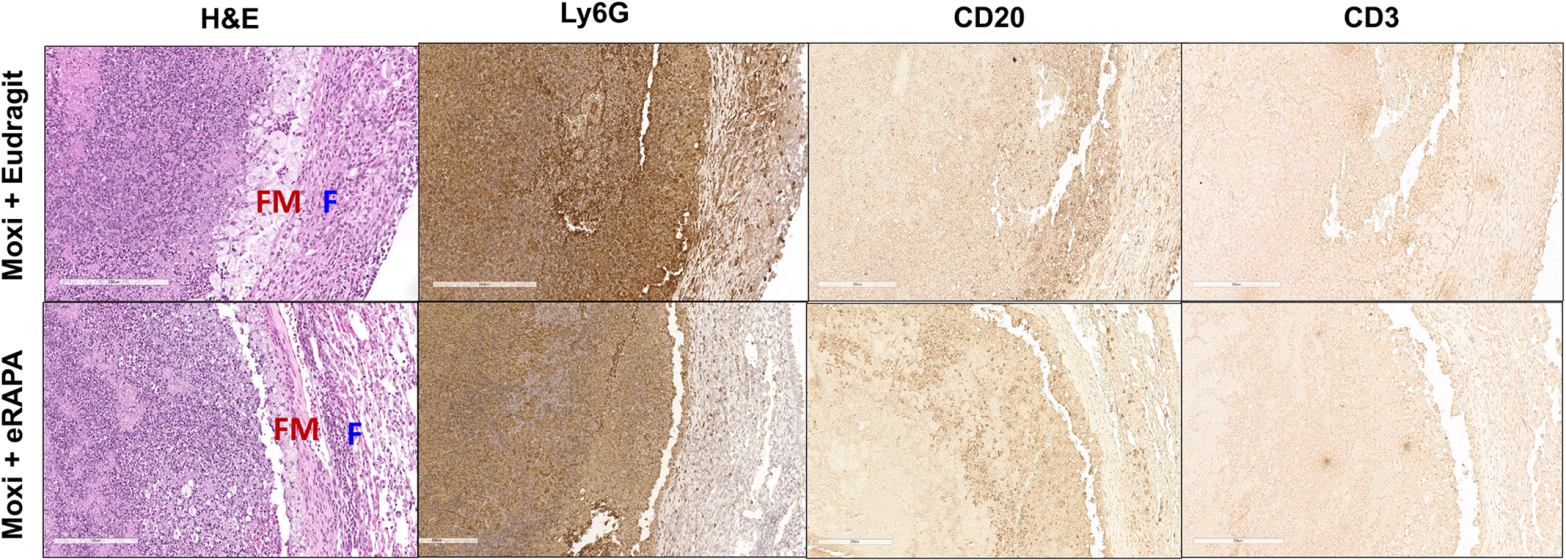
**Distinct cellular infiltrates surround necrotic lesions in eRAPA- and Eudragit-treated mice.** Mtb-infected C3HeB/FeJ mice were fed either Eudragit or eRAPA diets, along with oral gavage of moxifloxacin from week 7 to week 9 post infection. Formalin-fixed paraffin-embedded lung sections from these groups were then H&E stained or immunostained for Ly6G^+^ (neutrophils), CD20^+^ (B cells) and CD3^+^ (T cells). These sections were scanned using a Leica SCN-400 F whole-slide scanner up to 40× magnification. Five mice were included in each group. Representative images are shown at 20× magnification. Scale bars: 200 µm. F, fibroblasts; FM, foamy macrophages.

Representative necrotic lesions (Fig. S5) from both groups were closely evaluated to determine whether eRAPA adjunct treatment with moxifloxacin not only reduced the number of necrotic lesions but also modified them qualitatively. H&E staining of paraffin-embedded lung sections revealed that the necrotic lesions from both groups had a central acellular caseum that contained large numbers of neutrophil karyorrhectic debris and were well-defined circumscribed lesions (Fig. S5). The parenchymal area surrounding the lesion in the eRAPA-treated group appeared more normal compared to that from the Eudragit-treated group (Fig. S5). Higher magnification revealed that the necrotic lesions from both groups were surrounded by a ring of foamy macrophages (FM) and an outer layer of fibroblasts (F) (Fig. 6). Immunohistochemical staining was performed to further characterize the immune infiltrates surrounding the necrotic lesions. Examination of the large necrotic lesions from both groups showed that the necrotic lesion in moxifloxacin-treated mice on a Eudragit diet was surrounded by a rim of intact and necrotic neutrophils in the foamy macrophage and fibroblast layers (Fig. 6). In contrast, in the eRAPA plus moxifoxacin-treated group, intact neutrophils were seen around the lesion, albeit in significantly fewer numbers than observed in the rim from Eudragit plus moxifloxacin-treated mice. Lesions from both groups showed the presence CD20^+^ B cells, but no CD3^+^ T cells were discernible (Fig. 6).

**Fig. 7.**
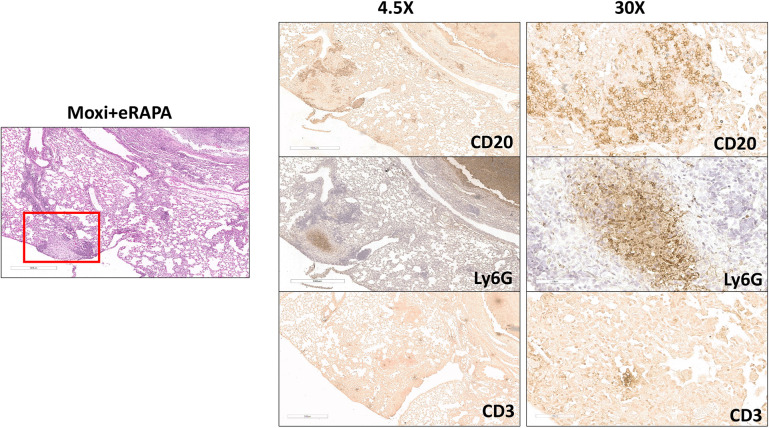
**Small necrotic lesions and B cell aggregates observed in eRAPA and moxifloxacin treated mice.** Standard H&E staining and immunostaining with anti-Ly6G, anti-CD20 or anti-CD3 antibodies were performed on formalin-fixed paraffin-embedded lung sections from Mtb Erdman infected C3HeB/FeJ mice that were fed an eRAPA diet during moxifloxacin monotherapy. Representative scanned images were captured to show a small necrotic lesion with B cell aggregates surrounding it, as indicated with the red square. These sections were scanned up to 40× using Aperio ImageScope. Five mice were included in this group. Scale bars: 500 µm (left and left column); 70 µm (right column).

**Fig. 8.**
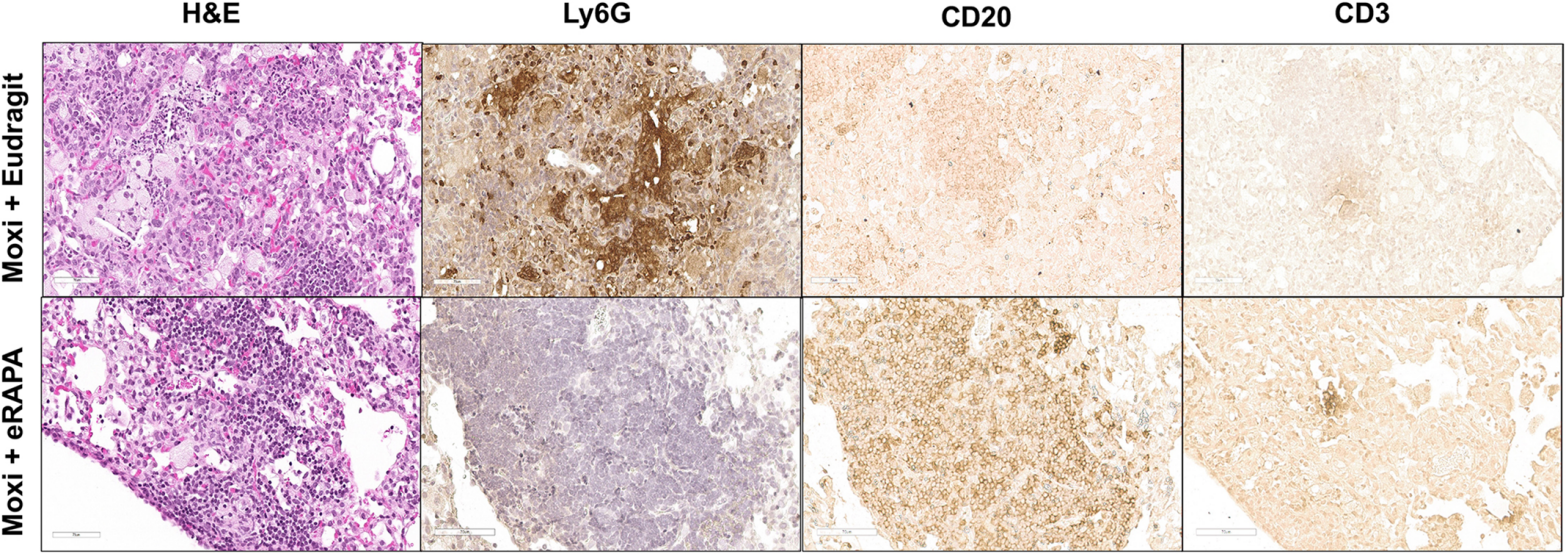
**Reduced neutrophil infiltration in lungs of eRAPA- and moxifloxacin-treated mice.** Formalin-fixed paraffin-embedded lung sections were obtained from Mtb Erdman-infected C3HeB/FeJ mice that were given either control diet or eRAPA (starting at week 7 post infection) along with moxifloxacin monotherapy. Standard H&E staining and immunostaining with anti-Ly6G, anti-CD20, or anti-CD3 antibodies were performed on these lung sections. Representative images were captured to show inflamed granulomatous areas in the lung. These sections were scanned up to 40× using the Leica SCN-400 F whole-slide scanner. All images are shown at 30× magnification. Five mice were included in each group. Scale bars: 70 µm.

Moxifloxacin- and eRAPA-treated mice also exhibited smaller necrotic lesions (Fig. 7); therefore, we next analyzed the staining pattern in this lesion type and found that it contained a central neutrophilic core with a surrounding fibrotic area and an outer rim of B cell aggregates (Fig. 7). Small indistinct CD3^+^ clusters were present within the B cell aggregates (Fig. 7, right CD3 panel). Similar small necrotic lesions were not apparent in Eudragit-treated mice. Examination of inflammatory regions outside of the necrotic lesions revealed a distinct composition of cells within the cellular aggregates. As seen in Fig. 8, the cellular aggregate in Eudragit plus moxifloxacin-treated mice included small numbers of CD20^+^ B and CD3^+^ T cells but contained numerous neutrophils, whereas in eRAPA plus moxifloxacin-treated mice the aggregate predominantly included CD20^+^ B cells and contained no neutrophils. Overall, the mice that received eRAPA adjunct therapy with moxifloxacin developed granulomas that appeared as tight well-contained cellular aggregates and showed minimal lung inflammation, even during chronic stages of Mtb infection.

## DISCUSSION

Autophagy-targeted HDTs are attractive as data from several studies indicate that activation of this pathway not only enhances bacterial killing (Watson et al., 2012; Gutierrez et al., 2004; Parihar et al., 2014; Stanley et al., 2014; Schiebler et al., 2015) but also dampens harmful inflammation (Castillo et al., 2012; Bonilla et al., 2013). The mTOR signaling pathway inhibits autophagy (reviewed by Kubota et al., 1968), and consequently mTOR inhibitors provide a host-directed therapeutic strategy to promote autophagy induction in Mtb infection. In this regard, the AMP-activated protein kinase (AMPK) stimulates autophagy by inhibiting mTOR phosphorylation through the tumor suppressor complex (TSC) (Gwinn et al., 2008; Inoki et al., 2003). Thus, metformin, which stimulates AMPK to mediate the inhibition of mTOR signaling, has been shown in the mouse model to restrict intracellular growth of Mtb and reduce immunopathology (Singhal et al., 2014).

In this study, we evaluated the performance of rapamycin, an mTOR inhibitor, in modulating bacterial burden and lung pathological disease during Mtb infection. The data presented here demonstrate that mice that received rapamycin alone or as an adjunct treatment with moxifloxacin had significantly less lung inflammation compared to those on a Eudragit diet or a moxifloxacin and Eudragit diet. Lung architecture significantly improved in mice on an eRAPA diet, as indicated by decreased cellular infiltration around necrotic lesions, the presence of discrete solid lesions and more open alveolar spaces. Although not statistically significant, a greater number of mice on an eRAPA diet had zero or fewer necrotic granulomas both at weeks 8 and 9 post infection, thus showing an overall reduced occurrence of these lesions compared to animals on an Eudragit control diet. Interestingly, eRAPA-treated mice exhibited B cell aggregates with a concomitant decrease in neutrophilic inflammation. Given that B cells regulate neutrophilia during Mtb infection (Kozakiewicz et al., 2013; Maglione et al., 2007), future studies should examine the mechanisms of how the suppression of inflammation by rapamycin can promote B cell aggregation, and how this contributes to the tempering of immunopathology. Initiation of rapamycin treatment in the chronic phase for 2 weeks had a better overall outcome in the mice, suggesting that the timing of rapamycin initiation and the duration of treatment is critical for its performance as an adjunct therapy. The C3HeB/FeJ model system will allow further interrogation of these variables along with rapamycin analogs. The model system will also provide a system to study whether modulating necrotic lesion development with rapamycin analogs will impact the evolution of bacterial drug resistance. Overall, the findings provide a strong basis for future studies to dissect mechanisms of necrotic lesion evolution and the impact of rapamycin and its analogs on this process.

Pulmonary TB can lead to a plethora of post-disease complications, including alteration of lung parenchyma, bronchiectasis and scarring of the lung (Ravimohan et al., 2018). Even after the successful completion of treatment, severe lung impairment can potentially impact the patient's quality of life (Meghji et al., 2020). Of note, is the complex interplay between co-morbid diseases, such as chronic obstructive pulmonary disease (COPD) and bronchiectasis with TB, which can further exacerbate the dysregulation of host immune responses and often lead to deleterious outcomes for the host (Byrne et al., 2015). A population-based cohort study indicated that the relative risk of developing active TB and subsequent risk of mortality in COPD patients was higher versus the general population (Inghammar et al., 2010). Previous history of TB can also play an important role in the natural course of COPD, as it has been reported that patients with past TB who were diagnosed with COPD died 5 years earlier compared to the patients without TB (Yakar et al., 2017). Additionally, treatment with inhaled corticosteroids in patients with COPD (Huang et al., 2020) has been associated with a significantly higher risk of TB, as shown by meta-analysis of clinical trials (Dong et al., 2014) and non-randomized studies (Castellana et al., 2019). Thus, the contribution and impact of pulmonary TB to the etiology of other chronic respiratory diseases, and vice versa, needs to be critically considered when designing treatment modalities in endemic TB areas.

Dampening excessive systemic inflammation and downstream tissue damage is by far the most important metric for improving outcomes for patients with active TB. Biological agents or repurposed drugs that can interfere with biologically relevant cellular checkpoints, can have a high potential to act as a ‘target-organ saving’ strategy (Zumla et al., 2015). Anti-inflammatory agents, such as vitamin D, phenylbutyrate, prednisolone and others, have been shown to play a role in improving sputum smear conversion rate and chest radiological appearance, making them noteworthy and viable HDT candidates (Hayford et al., 2020). Also of interest is the potentially beneficial role of non-steroidal anti-inflammatory drugs (NSAIDs) in mitigating disease severity and reducing lung inflammation when used as an adjunct treatment along with standard anti-TB drugs (Kroesen et al., 2017).

Published work has shown that treatment with rapamycin inhibits inflammation and airway hyper reactivity in the disease models of COPD and asthma, which is consistent with our findings (Zhang et al., 2019; Mushaben et al., 2011; Mitani et al., 2016). Here, we demonstrate in a mouse model that treatment with rapamycin during chronic infection noticeably ameliorates lung inflammation without deleterious effects on bacterial burden. However, there are several important issues that still need to be addressed for the application of mTOR inhibitors as a HDT against TB. Treatment with everolimus induced reactivation of latent TB in organ transplant patients (Guirao-Arrabal et al., 2016). It is important to understand how mTOR inhibitors (including everolimus) function as effective adjunct therapies in diverse clinical settings, as rapamycin analogs, including everolimus, are currently being investigated as HDTs against TB in phase II clinical trials (Kim et al., 2004). CC-11050 and everolimus were safe and reasonably well tolerated as adjunctive therapies for TB, and analysis of preliminary efficacy suggests they might also enhance the recovery of FEV_1_, a key measure of lung function and predictor of all-cause mortality (Wallis et al., 2021).

Pulmonary cavitation has been observed in Mtb aerosol-infected C3HeB/FeJ mice using serial computed tomography imaging (Ordonez et al., 2016), further indicating that with advanced imaging tools these mice could serve as a very helpful tool for studying TB progression, pathogenesis and cavitary disease. Thus, the key similarities with human disease make C3HeB/FeJ mice a valuable small animal model for studying the efficacy of anti-TB interventions in bacterial control and mitigation of disease pathology.

## MATERIALS AND METHODS

### Ethics statement

All animal experiments described in this study conform with the Rutgers University Biomedical Health Sciences and Institutional Animal Care and Use Committee (IACUC) Guidelines, as well as with National Institutes of Health and United States Department of Agriculture policies on the care and use of animals in research and teaching. Efforts were taken to ensure minimal animal pain and suffering, and when applicable, approved anesthesia methods were employed.

### Mice and aerosol infection

Female 6- to 8-week-old C3HeB/FeJ mice were purchased from the Jackson Laboratory (Bar Harbor, ME, USA) and infected with a low dose (∼50-100 CFU) of Mtb Erdman strain (Trudeau Institute, Saranac, NY, USA) using Glas-Col Full Body Inhalation Exposure. For all infections, the actual infection dose was determined by plating total lung homogenates from a minimum of three mice on Middlebrook 7H11 plates at 24 h after aerosol exposure, and CFUs were determined after 3-4 weeks incubation at 37°C. Mtb-infected mice were housed in the animal biosafety level 3 facility, and Rutgers University New Jersey Medical School IACUC guidelines were followed when handling the mice.

### Estimation of the bacterial burden from lung and spleen

Whole lungs and spleens were excised from the infected mice at the indicated time points and homogenized in PBS with 0.05% Tween 80 (PBST). Lung and spleen homogenates were plated on 7H11 agar in serial dilutions. CFUs from lung and spleen were determined after incubation at 37°C for 3-4 weeks.

### Rapamycin diet and moxifloxacin administration

We obtained eRAPA from Rapamycin Holdings (San Antonio, TX, USA). The control diet contained an equivalent amount of Eudragit S100 enteric polymer (Eudragit), the encapsulating polymer. Diet containing this Eudragit microencapsulated rapamycin was custom manufactured by TestDiet (St Louis, MO, USA). The eRAPA diets contained active rapamycin at either 14 ppm (for experiments presented in Fig. 3 to Fig. 8; Fig. S2) or 42 ppm (for experiments presented in Figs 1,2; Figs S1, S5) (Harrison et al., 2009). Mice were fed these eRAPA or Eudragit diets daily for the indicated time period. A solution of moxifloxacin (Sigma-Aldrich) in distilled water was prepared weekly and stored at 4°C. Moxifloxacin at a concentration of 100 mg/kg in distilled water was administered to mice by oral gavage for 5 days per week, at indicated time intervals.

### Cytokine and chemokine estimation in lung homogenates

Whole lungs from infected animals were homogenized in PBST. After homogenization, a fraction of the lung homogenate was treated with 2× protease inhibitor (Thermo Fisher Scientific) at the time of collection and frozen at −80°C. A mouse cytokine ultrasensitive immunoassay (mouse pro-inflammatory V panel, K15048D-2, Meso-Scale Discovery, Gaithersburg, MD, USA) was used to quantify cytokine and chemokine levels. The plates were read using an Meso Scale Discovery (MSD) detector (Sector Imager 2400, MSD, Gaithersburg, MD, USA). The assays are based on the principle of electrochemiluminescence sandwich ELISA. The calculations to establish calibration curves and determine analyte concentrations were carried out using the MSD Discovery Workbench analysis software.

### Histopathological assessment

Postmortem, lungs of Mtb-infected mice were perfused with sterile PBS and subsequently fixed in 4% paraformaldehyde for 7 days, followed by paraffin embedding. For histopathological analysis, 5- to 7-μm sections were cut and stained using a standard H&E protocol. A Leica SCN400 F whole-slide scanner (Experimental Pathology Research Lab, NYU Langone Health, NY, USA) was used for scanning histological sections, and images were analyzed using Aperio ImageScope. For the quantification of granulomatous inflammation in the lung section, Image-Pro Discovery Software was used to create a grid overlay onto each photomicrograph of H&E-stained lung section and numbers of points hitting areas of granulomatous infiltration were counted. Mosaic images were created using Surveyor software with Turboscan by 20× objective imaging. Histopathological evaluations were performed with blinding to the type of diet that the animals were on.

### Rapamycin measurement

Uninfected 6- to 8-week-old female C3HeB/FeJ mice were fed either Eudragit control or eRAPA diets for 17 days. Mice were then euthanized, and blood was collected via cardiac puncture in microtubes coated with heparin (Sarstedt 41.1393.105). Samples were stored at −80°C until shipment. Tubes were later shipped to the University of Texas Health Science Center Biological Psychiatry Analytical Lab for the estimation of rapamycin using liquid chromatography mass spectrometric analysis. This analysis was repeated twice in two independent tests.

### Flow cytometry

At indicated time points, lungs were perfused with 10 ml of PBS and then the middle and inferior lobes of the right lung were harvested. Lung tissue was digested in 2 mg/ml collagenase D (Roche) at 37°C for 30 min and 10 mM EDTA was added to halt the reaction. The digested tissue was then mashed through a 40-µm nylon filter using the plunger of a syringe. The single-cell suspension was then centrifuged at 250 ***g*** for 10 min and the cell pellet was subjected to red blood cell lysis using ammonium chloride potassium lysis buffer (Quality Biological). Live cell count was then determined using the Trypan Blue exclusion method. A quantity of 1×10^6^ live single cells from each sample were then surface stained with directly conjugated fluorochrome-labeled anti-mouse IgG CD4-V450 (clone RM4-5, BD Horizon), anti-mouse IgG CD8 Alexa Fluor 488 (clone 53-6.7, BD Pharmingen), anti-mouse IgG B220-PECF594 (clone RA3-6B2, BD Pharmingen), anti-mouse IgG CD11b-APC Cy7 (clone M1/70, BD Pharmingen), anti-mouse IgG Ly6G-BV605 (clone 1A8, BD Pharmingen), anti-mouse IgG CD11c-AF700 (clone HL3, BD Pharmingen) and anti-mouse IgG Ly6C-APC (clone HK1.4, BioLegend) antibodies at a 1:50 dilution in FACS Buffer (PBS, 10% FBS, 0.1% NaN_3_ sodium azide). Following surface staining, cells were fixed in 4% paraformaldehyde and then acquired on a BD Fortessa flow cytometer. Analysis was performed using FlowJo software (Tree Star). Gating for the myeloid population was based on fluorescence minus one control.

### Immunohistochemistry

Tissue sections were deparaffinized in xylene and hydrated with ethanol gradations and water. Ly6G, CD20 and CD3 epitopes were retrieved by the heat-induced antigen retrieval method using 10 mM citrate buffer (pH 6). Endogenous peroxidase activity was blocked using 0.3% hydrogen peroxide and then subsequently blocked with 1× Power Block (BioGenex). Sections were then incubated with primary goat anti-mouse IgG CD20 antibody (clone M-20, Santa Cruz Biotechnology), rat anti-mouse IgG Ly6G antibody (clone 1A8, BioLegend) and rat anti-mouse IgG CD3 antibody (clone 17A2, BioLegend) at a 1:100 dilution overnight at 4°C in Power Block. Sections were then washed with PBS-0.05% Tween 20 and incubated with biotinylated secondary antibodies (goat anti-rat IgG, BD 559286; rabbit anti-goat IgG, BA-5000, Vector Laboratories) at a 1:100 dilution for 45 min. Streptavidin horseradish peroxidase (BioGenex, HK3305K) was used to label the secondary antibody for immunodetection by DAB chromogen (Biogenex). After counterstaining with Mayer's Hematoxylin (BioGenex), the samples were dehydrated with ethanol gradations, dipped in xylene and mounted using Cytoseal (Thermo Scientific).

### Statistics

GraphPad Prism software was used to perform statistical analyses. An unpaired *t*-test was used to compare two groups. One-way ANOVA was performed to determine the statistical significance for more than two groups. In all experiments, *P*<0.05 was considered statistically significant.

## Supplementary Material

Supplementary information
