# Supplementary material for: Rapamycin modulates pulmonary pathology in a murine model of Mycobacterium tuberculosis infection
Source: Dis Model Mech. 2021 Oct 26;14(10):dmm049018. doi: 10.1242/dmm.049018 (PMC8560501; doi:10.1242/dmm.049018)
Supplement: Supplementary information [file dmm-14-049018-s1.pdf]

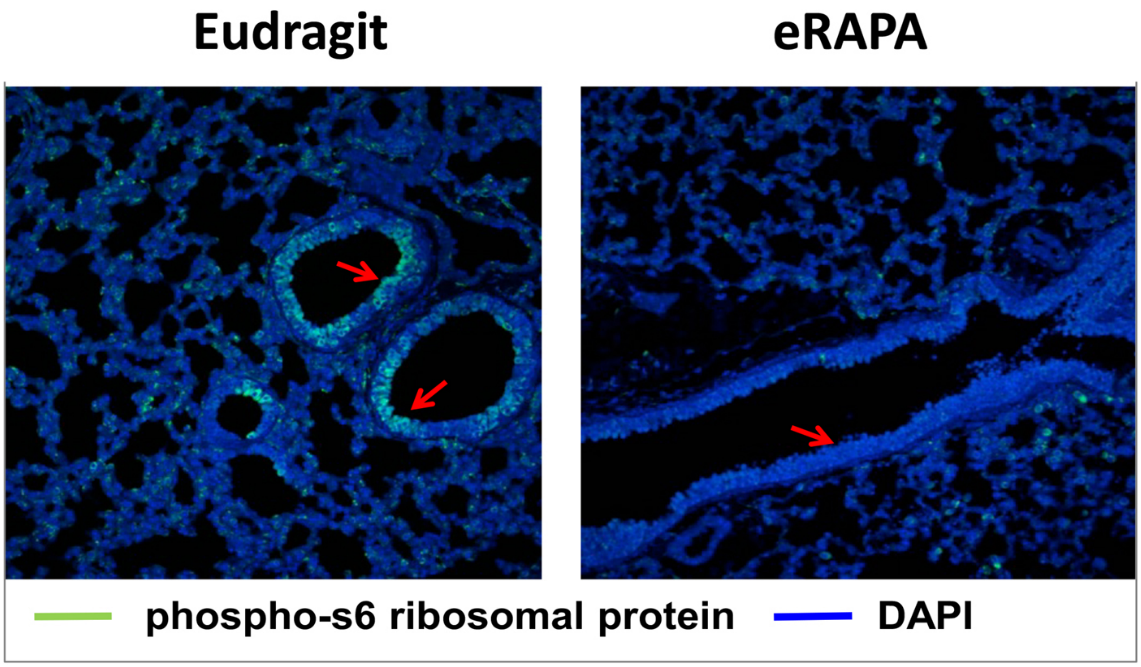

Fig. S1. Treatment with eRAPA leads to reduced expression of phospho-S6 ribosomal protein in alveolar bronchiole epithelial cell lining.
C3HeB/FeJ mice infected with low dose of Mtb Erdman were either fed eudragit or eRAPA diets, starting at week 2 post-infection. The animals were then euthanized 2 weeks after the initiation of treatment regimen. PhosphoS6 protein (Santa Cruz, sc-293144) was detected using immunofluorescence in formalin fixed paraffin embedded lung sections of these eudragit (control) or eRAPA treated mice. Nuclear counterstaining was done using DAPI. Images were captured on Nikon A1R confocal laser scanning microscope and analyzed using NIS Elements. This image is representative of 5-6 animals in each group.

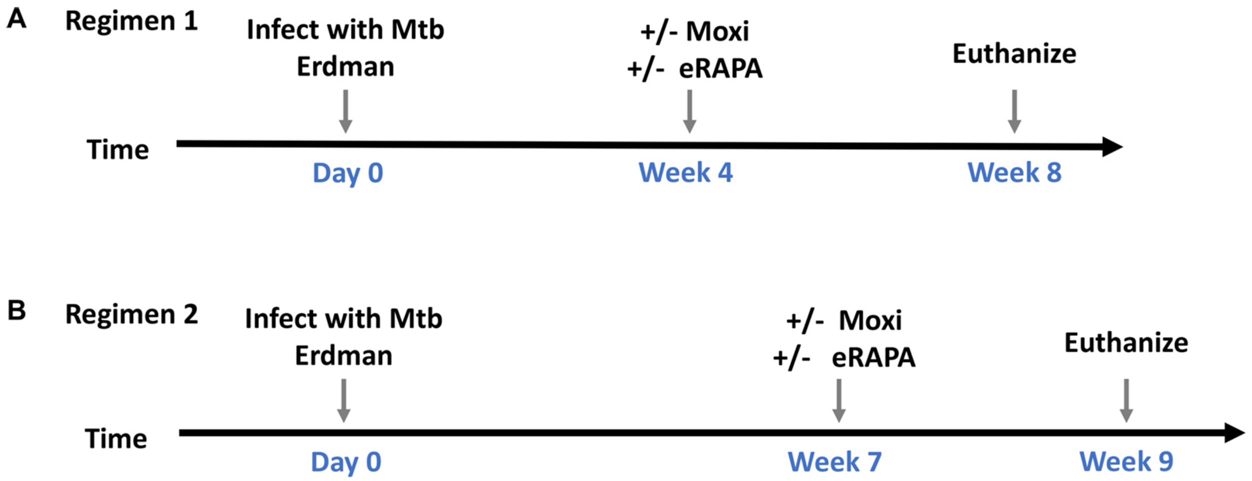

Fig. S2. Experimental scheme.
Schematic overview of the timeline for Mtb infection, eRAPA treatment and tissue harvest for moxifloxacin monotherapy.

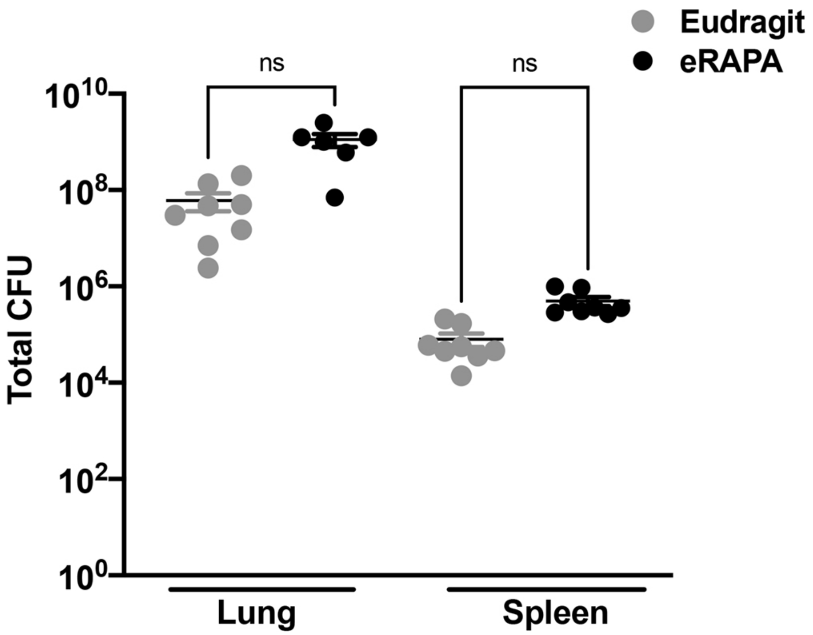

Fig. S3. Bacterial burden in Mtb-infected animals receiving eudragit or eRAPA at 4 weeks following Mtb infection.
C3HeB/FeJ mice were infected with low dose of Mtb Erdman. Four weeks post Mtb infection, mice were either fed eudragit or eRAPA diets. The animals were sacrificed 4 weeks after the initiation of treatment regimen. Whole lungs and spleens were homogenized for bacterial burden estimation. Six to eight animals were included in each group. Data are presented as mean ± standard errors of the means. Statistical significance was calculated using one-way ANOVA with Kruskal Wallis test.

Disease Models & Mechanisms • Supplementary information

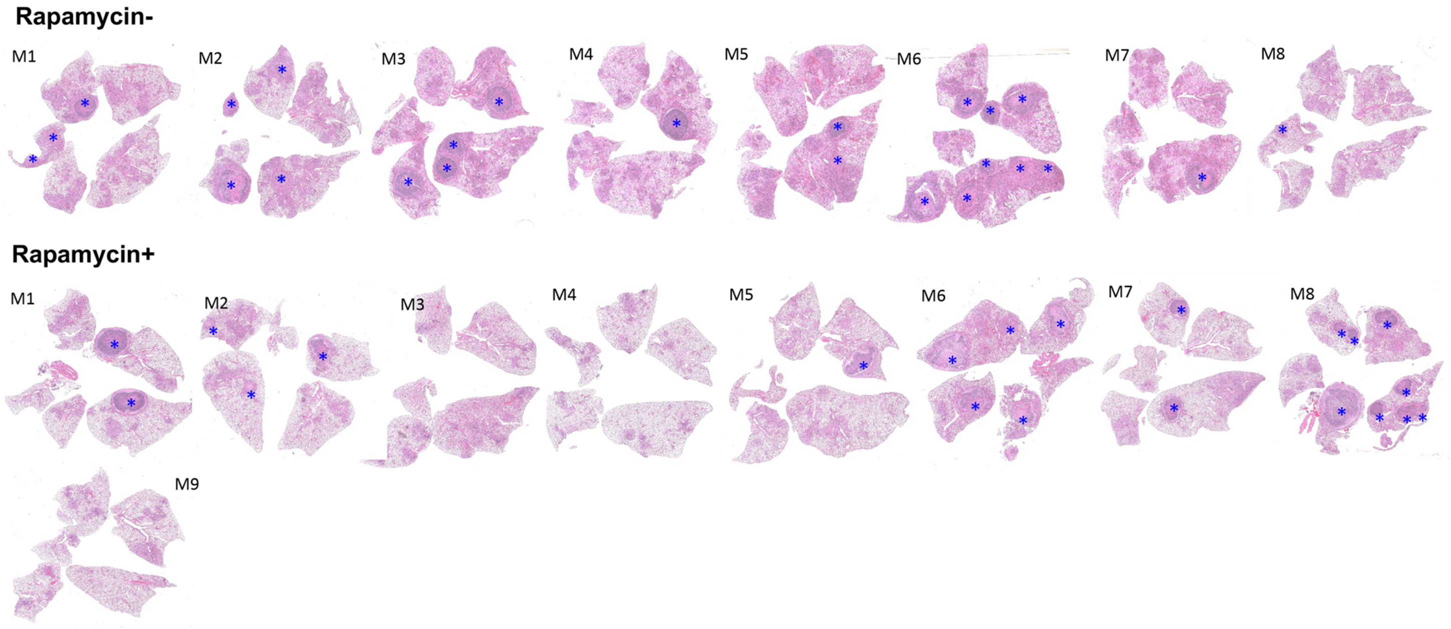

Fig. S4. eRAPA delivered by oral gavage leads to significant changes in histopathology. At week 7 post Mtb infection, C3HeB/FeJ mice were given eRAPA in orange juice at a daily dose of 0.04mg for two weeks, via oral gavage. All lung lobes processed for histology. H&E stained sections were scanned up to 40X using the Leica SCN-400 F whole slide scanner. Necrotic lesions are marked with blue asterisk and each group had 8 to 9 animals.

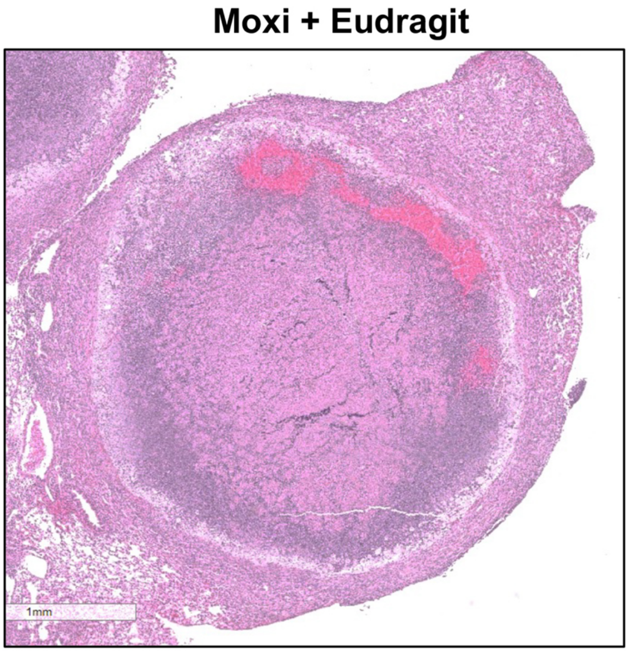

Fig. S5. H&E stained sections comparing necrotic lesions from eudragit and moxifloxacin versus eRAPA and moxifloxacin groups.
Representative scanned H&E images of areas in and around necrotic lesions of lung tissue sections of mice from eudragit and eRAPA treated groups at week 9 postinfection.
The scale is 1 mm, and five mice were included in each group.

Table S1. Estimation of rapamycin in murine serum samples

Test	Volume	Rapamycin (ng/mL)
**Test I**		
**Eudragit**	50 µl	< 1.56
	50 µl	no sample
	50 µl	< 1.56
	50 µl	< 1.56
**Rapamycin**	50 µl	188
	50 µl	170
	50 µl	201
	50 µl	149
**Rapamycin mean (+/-SD)**		177 (22.58)
**Test II**		
**Eudragit**	50 µl	< 1.56
	50 µl	< 1.56
	50 µl	< 1.56
	50 µl	< 1.56
**Rapamycin**	50 µl	217
	50 µl	254
	50 µl	225
	50 µl	237
**Rapamycin mean (+/-SD)**		233 (16.09)

Note: values <1.56 are below detectable limit.
